# Cytochrome P450 Epoxygenase-Dependent Activation of TRPV4 Channel Participates in Enhanced Serotonin-Induced Pulmonary Vasoconstriction in Chronic Hypoxic Pulmonary Hypertension

**DOI:** 10.1155/2020/8927381

**Published:** 2020-01-22

**Authors:** Yang Xia, Lexin Xia, Zhou Jin, Rui Jin, Omkar Paudel, James S. K. Sham

**Affiliations:** ^1^Department of Respiratory and Critical Care Medicine, Second Affiliated Hospital of Zhejiang University School of Medicine, Hangzhou, Zhejiang 310009, China; ^2^Division of Pulmonary and Critical Care Medicine, Johns Hopkins School of Medicine, Baltimore, MD 21224, USA

## Abstract

Transient receptor potential vanilloid 4 (TRPV4) is a multi-functional non-selective channel expressed in pulmonary vasculatures. TRPV4 contributes to serotonin- (5-HT-) induced pulmonary vasoconstriction and is responsible in part for the enhanced 5-HT response in pulmonary arteries (PAs) of chronic hypoxia mice. Epoxyeicosatrienoic acid (EET) is an endogenous agonist of TRPV4 and is known to regulate vasoreactivity. The levels of EETs, the expression of cytochrome P450 (CYP) epoxygenase for EET production, and epoxide hydrolase for EET degradation are altered by chronic hypoxia. Here, we examined the role of EET-dependent TRPV4 activation in the 5-HT-mediated PA contraction. In PAs of normoxic mice, inhibition of TRPV4 with a specific inhibitor HC-067047 caused a decrease in the sensitivity of 5-HT-induced PA contraction without affecting the maximal contractile response. Application of the cytochrome P450 epoxygenase inhibitor MS-PPOH had no effect on the vasoreactivity to 5-HT. In contrast, inhibition of CYP epoxygenase or TRPV4 both attenuated the 5-HT-elicited maximal contraction to a comparable level in PAs of chronic hypoxic mice. Moreover, the inhibitory effect of MS-PPOH on the 5-HT-induced contraction was obliterated in PAs of chronic hypoxic *trpv4^−/−^* mice. These results suggest that TRPV4 contributes to the enhanced 5-HT-induced vasoconstriction in chronic hypoxic PAs, in part via the CYP-EET-TRPV4 pathway. Our results further support the notion that manipulation of TRPV4 function may offer a novel therapeutic strategy for the treatment of hypoxia-related pulmonary hypertension.

## 1. Introduction

Chronic hypoxic pulmonary hypertension (CHPH), which belongs to the Group 3 in the pulmonary hypertension classification [1], can be instigated by sustained exposure to hypoxia. Increasing evidence indicates that nonselective cation channels affect intrinsic changes in ionic balance and Ca^2+^ homeostasis in the pulmonary arterial smooth muscle cells and play pivotal roles in acute [2, 3] and prolonged hypoxic responses [4–7].

Transient receptor potential (TRP) channels are a set of nonselective cation channels containing seven protein families [8]. TRPV4, serving as an osmo-mechanosensitive channel, is widely expressed and functioning in both systemic and pulmonary vasculatures [9] and is gated by numerous stimuli including moderate heat, shear stress, osmotic, chemical stimuli, and the endogenous agonist, epoxyeicosatrienoic acids (EETs) [8, 10–18]. Cytochrome P450 (CYP) epoxygenases, especially CYP 2 group, metabolize membrane arachidonate to generate EETs [19]. In systematic vasculatures, EET-induced TRPV4 activation causes potent vasodilating effect [14]. Several mechanisms have been proposed: (1) activation of calcium-activated K^+^ channels by the diffusion of endothelial-derived EETs to vascular smooth muscle [14]; (2) endothelial TRPV4 activation opens endothelial small and intermediate conductance Ca^2+^-activated K^+^ channels, resulting in direct coupling of the endothelium and smooth muscle or the accumulation of K^+^ in the extracellular space to hyperpolarize the smooth muscle [20]; and (3) TRPV4 coupled with ryanodine receptors and BK_Ca_ channels to elicit smooth muscle hyperpolarization and arterial dilation via Ca^2+^-induced Ca^2+^ release in response to putative EETs [14, 15, 21–23].

In the lung, TRPV4 channels are distributed in human bronchial epithelial cells, airway smooth muscle cells, endothelial cells, and vascular smooth muscle cells in pulmonary arteries (PAs) [16]. They are involved in multiple physiological functions. TRPV4 is reported to play many different roles in the regulation of cell volume, vasomotor tone, endothelial mechanosensation, thermosensing, and vascular/epithelial permeability [13]. In contrast to the vasodilatory effect in systematic vasculatures, TRPV4, in pulmonary vasculature, contributes to vasoconstriction [24]. We have previously found that chronic hypoxia (CH) upregulates the expression of TRPV4 in pulmonary arteries, which results in elevated myogenic tone, intracellular calcium ([Ca^2+^]_i_), and vascular remodeling [25]. TRPV4 regulates serotonin- (5-HT-) induced Ca^2+^ response in normoxia [24, 26]. More importantly, in chronic hypoxia, increased 5-HT-induced maximal contraction in PAs is partially reversed by TRPV4 antagonist [24]. In concordance, enhanced 5-HT-induced contraction is significantly reduced in PAs of hypoxic *trpv4*^−/−^ mice [24]. The development of hypoxia-induced pulmonary hypertension and pulmonary vascular remodeling is also delayed and suppressed in *trpv4*^−/−^ mice [25]. On the other hand, chronic hypoxia upregulates CYP epoxygenases expression [27], downregulates the soluble epoxide hydrolase [28], increases the TRPV4 agonist EETs production, and facilitates the acute hypoxic pulmonary vasoconstriction (HPV) and chronic hypoxia-induced pulmonary vascular remodeling [27, 28]. Moreover, blockage of soluble epoxide hydrolase enhances hypoxic pulmonary vasoconstriction, supporting the role of EETs in acute hypoxic response [29, 30]. However, whether CYP-EET-TRPV4 signaling pathway is involved in 5-HT-elicited PA contraction in normal condition and in chronic hypoxia has not been studied. Hence, we hypothesize that CYP-EET regulates the agonist-induced vasoconstriction through TRPV4 activation in pulmonary hypertension. In the present study, we aim to test this hypothesis by using the currently available selective antagonists and *trpv4*^−/−^ mice to probe the contribution of CYP-EET-TRPV4 in agonist-induced contraction in PAs of normal and CHPH mice.

## 2. Materials and Methods

### 2.1. Chronic Hypoxic Exposure

Male *trpv4^−/−^* mice and wild-type (WT) mice were age-matched (C57BL/6J; 8 weeks old). The mice are kind gifts from Dr. Wolfgang Liedtke's lab, Duke University. The generation of *trpv4^−/−^* mice have been previously described [31]. The mice were placed in a hypoxic chamber and exposed to hypoxia (10% O_2_) for 3-4 weeks to induce hypoxic pulmonary hypertension as described previously [24]. Control mice were housed in the same condition but exposed to room air.

### 2.2. Isolation and Isometric Tension Measurement of PAs

The mice pulmonary arteries were isolated, cut into segments, de-endothelialized by gentle rubbing of the lumen with a moose hair, and placed in Krebs solution which contains the following (in mM): 118 NaCl, 4.7 KCl, 0.57 MgSO_4_, 1.18 KH_2_PO_4_, 25 NaHCO_3_, 10 dextrose, and 1.25 CaCl_2_ as previously described [6, 24]. PA rings were then fixed on a wire myograph chamber with two stainless steel wires, filling with 16% O_2_ plus 5% CO_2_ gassed modified Krebs solution. Isometric tension development was recorded. The resting tension was set at the levels equivalent to 15 mmHg or 25 mmHg for normoxic and hypoxic mice, respectively. After a 60-minute equilibration, PA rings were exposed to 60 mM KCl to establish maximum contraction and to phenylephrine (PE, 1 *μ*M) followed by ACh (10 *μ*M) to verify disruption of endothelium. The active tension induced by TRPV4 agonist was normalized to maximum contraction generated by 60 mM KCl.

### 2.3. Chemicals and Drugs

HC-067047, 5-HT, MS-PPOH, and other chemicals were purchased from Sigma Chemical (St. Louis, MO). Stock solutions of HC-067047 and MS-PPOH were prepared in DMSO and diluted 1 : 1,000 in 2 mM Ca^2+^-Tyrode solution.

### 2.4. Statistical Analysis

All data in the article are represented as means ± SE. Three-parameter logistic model was applied for concentration-response curves as preciously described [24] (equation (1)), where *R* stands for the normalized developed tension, *E*_max_ stands for the maximal response, EC_50_ stands for the effective concentration for 50% response, and *b* stands for the slope factor.

(1) $$\begin{array}{c} R=\frac{E_{\max}}{1+{\left(\left[A\right]/{\text{EC}}_{50}\right)}^{b}}. \end{array}$$

Statistical significance (*P* < 0.05) of the changes was compared with paired or unpaired Student's *t* tests or by one- or two-way ANOVA with Bonferroni's post hoc test, wherever applicable.

## 3. Results

### 3.1. The Effect of HC-067047 and MS-PPOH in 5-HT-Induced PA Constriction in Normoxia

**Figure 1:**
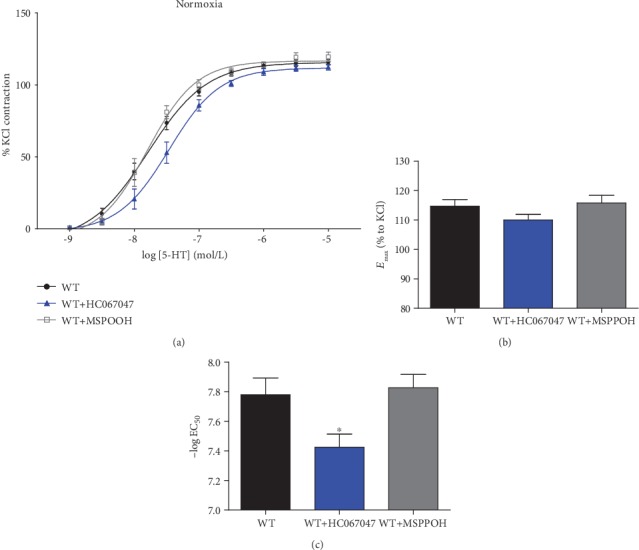
The effect of HC-067047 and MS-PPOH in normoxic WT mice. (a) Cumulative concentration-response curve of 5-HT, in PAs of WT mice with vehicle, MS-PPOH preincubation, and with HC-067047 preincubation, respectively. Data are expressed as a percentage of K^+^-induced contractile responses (60 mM). (b, c) Bar graph showing the contractile response of normoxic PAs of WT mice, WT mice with HC-067047 preincubation and WT mice with MS-PPOH preincubation to serotonin. Mean values of maximal response (*E*_max_) and −log EC_50_ is derived from experiments shown in (a). EC_50_ and *E*_max_ were calculated from each individual PA using the 3-parameter logistic equation described in Materials and Methods. ^∗^*P* < 0.05.

To evaluate the contribution of EETs and the downstream TRPV4 in 5-HT-elicited PA constriction in normoxia, vascular tension was measured by wire myograph in the absence or presence of the CYP epoxygenase inhibitor MS-PPOH and the TRPV4 inhibitor HC-067047. Application of MS-PPOH caused no significant change of *E*_max_ (Figure 1(b)) or EC_50_ (Figure 1(c)) in PAs of normoxic WT mice. Consistent with our previous finding [24], inhibition of TRPV4 with HC-067047 had no effect on E_max_ (Figure 1(b), *n* = 6), but it caused a right shift of the concentration-response curve (control: 7.780 ± 0.1134 vs. HC-067047: 7.425 ± 0.0884, *P* < 0.05), suggesting a decrease in sensitivity to 5-HT after TRPV4 inhibition. These results demonstrate that in normoxia, CYP expoxygenase-dependent EETs are not involved in 5-HT-induced PA contraction, while TRPV4 exhibits modest effect on 5-HT-generated PA constriction.

### 3.2. Contribution of CYP-EET-TRPV4 in 5-HT-Induced PA Constriction in Chronic Hypoxia

**Figure 2:**
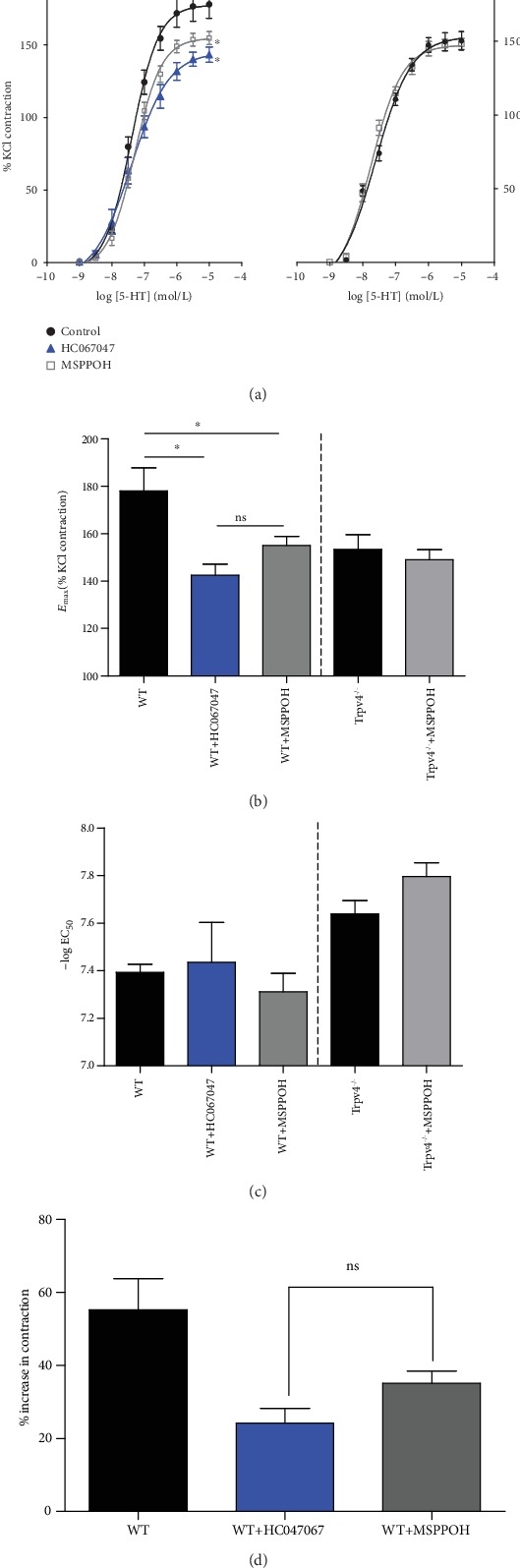
The effect of HC-067047 and MS-PPOH in WT and *trpv4^−/−^* mice in chronic hypoxia. (a) Cumulative concentration-response curve of 5-HT in PAs of WT mice and *trpv4^−/−^* mice with MS-PPOH preincubation and HC-067047 preincubation. Data are expressed as a percentage of K^+^-induced contractile responses (60 mM). (b, c) Bar graph showing the contractile response of hypoxic PAs of WT mice and *trpv4^−/−^* mice to serotonin with HC-067047 or MS-PPOH preincubation. Mean values of maximal response (*E*_max_) and −log EC_50_ is derived from experiments shown in (a). EC_50_ and *E*_max_ were calculated from each individual PA using the 3-parameter logistic equation described in Materials and Methods. (d) Bar graph showing percent inhibition of K^+^-induced contraction by HC-067047 and MS-PPOH in WT mice. ^∗^*P* < 0.05; ns: nonsignificant.

Consistent with previous findings, 5-HT-induced PA contraction was significantly potentiated in endothelium-denuded CH PAs (*n* = 10) compared to normoxic PAs (*E*_max_: 178.1 ± 9.767% vs. 113.2 ± 1.833%, *P* < 0.01). Significant suppression of the enhanced 5-HT response was observed after CYP epoxygenase inhibitor, MS-PPOH treatment (*n* = 6, *P* < 0.05), and the inhibitory effect was similar to PAs treated with TRPV4 blocker, HC-067047 (*n* = 6, nonsignificant). Moreover, 5-HT-activated maximum contractile response in genetic deletion of *trpv4* (*n* = 10) was identical to that activated in WT PAs after TRPV4 inhibition (*trpv4^−/−^E*_max_: 153.4 ± 6.207% vs. WT+ HC-067047: 142.5 ± 4.603%, Figure 2(a)). Blockage of CYP epoxygenase inhibitor showed no effect on neither *E*_max_ nor EC_50_ in *trpv4* null mice (*n* = 6, Figures 2(b) and 2(c)).

In extension, we further evaluated the percentage increase in maximal response to 5-HT. Basically, CH caused 55% increase in maximum response to serotonin (Figure 2(d)). Of note, the percent enhancement was dramatically attenuated in HC-067047-treated and MS-PPOH-treated PAs of CH WT mice. Moreover, the *E*_max_ (HC-067047: 24.24 ± 4.013% vs. MS-PPOH: 35.16 ± 3.336%, *P* > 0.05) and −log EC_50_ (HC-067047: 7.436 ± 0.1673 vs. MS-PPOH: 7.311 ± 0.07788, *P* > 0.05) of 5-HT-induced contractions were comparable in PAs of CH mice treated with HC-067047 and MS-PPOH. Most importantly, MS-PPOH did not caused additional inhibition in 5-HT induced PA constriction in CH *trpv4^−/−^* mice (Figure 2(a)), indicating that the MS-PPOH-dependent inhibitory effect on 5-HT-induced contraction in CH WT mice was mediated specifically through the TRPV4 pathway. Collectively, these results clearly suggest that CYP-EET-TRPV4 is involved in the enhanced 5-HT-induced PA contraction in CH.

## 4. Discussion

Enhanced vasoreactivity is a fundamental pathogenic mechanism for CHPH. In present study, we used pharmacological tools and *trpv4* gene-deleted mouse models to test the hypothesis that the CYP-EET-TRPV4 pathway regulates the agonist-induced vasoconstriction in pulmonary hypertension. The major findings are as following: (1) in normoxia, inhibition of TRPV4 with a specific inhibitor HC-067047 caused a decrease in the sensitivity of 5-HT-induced PA contraction, as consistent with previous reports [24], while CYP epoxygenase inhibitor MS-PPOH did not affect vasoreactivity to 5-HT; (2) in chronic hypoxia, blockage of CYP epoxygenase or TRPV4 both attenuated 5-HT-elicited PA contraction to a similar level. More importantly, the inhibitory effect of MS-PPOH on 5-HT induced PA contraction was is not observed in *trpv4^−/−^* mice PA. These results suggest that the CYP-EET-TRPV4 pathway is associated with 5-HT-dependent pharmacomechanical coupling in pulmonary hypertension.

A wealth of data shows that 5-HT plays a critical role in the hypoxia-induced pulmonary hypertension. Pulmonary hypertension is associated with increased plasma 5-HT [32], upregulated 5-HT1B and 5-HT2B receptors [33–35] and 5-HT transporter [36–38], and enhanced 5-HT-induced pulmonary vasoconstriction [33, 36, 39–41]. The involvement of CYP-EET-TRPV4 pathway in the enhanced 5-HT-induced vasoconstriction in CH pulmonary hypertension is supported by several lines of evidence. First, 5-HT has implication for the activation of TRPV4 in PASMCs. It has been shown that 5-HT activates ion current and Ca^2+^ influx which resemble TRPV4 activation in PASMCs; and the serotonin-activated current and Ca^2+^ signal could be inhibited by TRPV4 inhibitors and the CYP epoxygenase inhibitor 17-ODYA [42]. Second, TRPV4 expression is increased in PASMCs of CH rats [5, 25] providing an enhanced Ca^2+^ influx pathways for pulmonary vasoconstriction. Third, EETs metabolized from arachidonic acid (AA) by CYP epoxygenase are important endogenous agonists for TRPV4 activation [43]. Hypoxia exposure causes an increase of CYP expression and a resultant excessive production of endogenous EETs in the lung tissues was in line observed [27] and decreased the expression of soluble epoxide hydrolase, attenuating the metabolism of EETs into the inactive form [28] Fourth, 5-HT is known to regulate CYP enzymes' expression to cause an increase in CYP1A1 expression in the intestinal epithelial cells and CYP1B1 expression in PASMCs via the serotonin transporters [44–46]. Hence, it is most likely that part or all of the components on CYP-EET-TRPV4-serotonin pathway contribute to the enhanced vasoreactivity in the PAs of CH animals, including 5-HT receptor upregulation, the increased CYP epoxygenase expression and EET production, and the upregulation of TRPV4 expression. This is consistent with our present observations that CYP-EET-TRPV4 pathway only has minimal effect on 5-HT-induced vasoconstriction in normoxic endothelium-free PAs. By contrast, the CYP-EET-TRPV4 pathway exhibits a significant influence in the elevated vasoreactivity in chronic hypoxia. Collectively, our observations vividly portray the pivotal role of the CYP-EET-TRPV4 pathway in the regulation of pulmonary vascular functions.

It has to be pointed that the enhanced pulmonary vasoconstriction is not completely inhibited by HC-067047 or MS-PPOH, because there are signaling mechanisms and channels, other than TRPV4, are participating in the 5-HT-induced pulmonary vasoconstriction. We have previously shown that TRPC6 is critically involved in 5-HT-generated contractile responses in PA under CH, and TRPC1 also contributes in part to the enhanced vasoreactivity to 5-HT in CH [6]. Moreover, other channels including TRPV1 [47], voltage-gated K+ channels [48], and calcium-activated chloride channel [49, 50] also participate in 5-HT-induced pulmonary vasoconstriction.

There are several limitations of our study. First, our results only reflect observations in male animals, and gender differences have not been determined. It has been shown that hyperpolarization induced by EETs in systemic arteries is more pronounced in female than in male, and the effect of sex in CYP epoxygenase-related vasoresponses is crucial. Second, our study here focuses specifically on the functional aspect of pulmonary vascular reactivity to examine our hypothesis of the participation of the CYP-EET-TRPV4 signaling pathway in the enhanced 5-HT-induced response in PA of chronic hypoxic mice. Future studies of additional cellular and molecular biology experiments are needed to provide a complete evaluation of this interesting observation of change in the modality of 5-HT-mediated signaling transduction.

Our present results clearly identified the differential role of CYP-EET-TRPV4 pathway in 5-HT-induced PA contraction under normoxic condition and CHPH and provide the supportive evidence that EETs participate in 5-HT-induced pulmonary vasoconstriction via the activation of TRPV4 channels in chronic hypoxia. Of note, the physiological and pathological roles of CYP-EET-TRPV4 pathways in PA remodeling and PASMC proliferation and migration in CHPH remain important for further investigation. Targeting TRPV4 may offer a novel therapeutic strategy for the treatment of hypoxia-related pulmonary hypertension.

## Data Availability

The data used to support the findings of this study are included within the article.
